# 840. Clinical Characteristics of Patients Living with HIV Hospitalized for COVID-19

**DOI:** 10.1093/ofid/ofab466.1036

**Published:** 2021-12-04

**Authors:** Nimra Chaudhry, Eris Cani, Tae Park, Cosmina Zeana, Paul Kelly, Haozhe Sun

**Affiliations:** BronxCare Health System, Brooklyn, New York

## Abstract

**Background:**

Limited data exists regarding the impact of coronavirus disease 2019 (COVID-19) on people living with human immunodeficiency virus (PLWH). The purpose of the study was to compare the clinical outcomes of patients hospitalized with COVID-19 and HIV versus those without HIV.

**Methods:**

This was a retrospective, cohort study of adult patients admitted with confirmed COVID-19 from March 1^st^ to May 30^th^ 2020 at an urban hospital in New York City. Data collected included demographics, past medical history, HIV status, baseline laboratory values, treatment and outcomes such as length of stay, mechanical ventilation, patient disposition at discharge, and in-hospital mortality. Fisher’s exact test was used to compare categorical values and a t-test was used to compare continuous values.

**Results:**

Out of 983 patients, 6.9% were PLWH and 93.1% were HIV-negative. The average age in both groups was 61 vs. 62 years, respectively. There were more male patients in the PLWH than the non-HIV group (76.8% vs. 58.6%). Majority of PLWH were Black (49.3%). Forty-seven percent of PLWH were mechanically ventilated versus 33.3% of the non-HIV group. The most common comorbidity in both groups was hypertension (82.4% vs. 72.6%). When compared to HIV-negative patients, PLWH had a higher rate of kidney disease (72.1% vs. 53.6%, p=0.0086), chronic obstructive pulmonary disease (41.2% vs. 14.5%, p=0.0001), liver disease (45.6% vs. 11.5%, p=0.0001) and current smoking (14.3% vs. 5.8%, p=0.0103). In PLWH, 70.6% of patients were on an integrase-based regimen. Fifty-three percent of PLWH had a CD4 count of > 200 cells/mm^3^ and 35.3% had an undetectable viral load (< 20 copies/mL). Unadjusted hospital mortality was 51.4% in PLWH and 36.2% in the non-HIV cohort (p=0.0089). The average length of hospital stay was 9.1 days vs. 8.4 days in PLWH versus the non-HIV group (p=0.4493). More patients were discharged to a nursing home in the non-HIV group vs. PLWH (37.8% vs. 14.3%, p=0.0001).

**Conclusion:**

Hospitalized patients with COVID-19 and HIV had a higher in-hospital mortality compared to those without HIV during the first COVID wave in New York City.

**Disclosures:**

**All Authors**: No reported disclosures

